# INMEX—a web-based tool for integrative meta-analysis of expression data

**DOI:** 10.1093/nar/gkt338

**Published:** 2013-06-12

**Authors:** Jianguo Xia, Christopher D. Fjell, Matthew L. Mayer, Olga M. Pena, David S. Wishart, Robert E. W. Hancock

**Affiliations:** ^1^Department of Microbiology and Immunology, University of British Columbia, Vancouver, V6T 1Z3, Canada, ^2^Centre for Microbial Diseases and Immunity Research, University of British Columbia, Vancouver, V6T 1Z4, Canada, ^3^James Hogg Research Centre, University of British Columbia, Vancouver, V6Z 1Y6, Canada, ^4^Department of Computing Science, University of Alberta, T6G 2E8, Canada, ^5^Department of Biological Sciences, University of Alberta, T6G 2E8, Canada and ^6^Wellcome Trust Sanger Institute, Hinxton, CB10 1SA, UK

## Abstract

The widespread applications of various ‘omics’ technologies in biomedical research together with the emergence of public data repositories have resulted in a plethora of data sets for almost any given physiological state or disease condition. Properly combining or integrating these data sets with similar basic hypotheses can help reduce study bias, increase statistical power and improve overall biological understanding. However, the difficulties in data management and the complexities of analytical approaches have significantly limited data integration to enable meta-analysis. Here, we introduce integrative meta-analysis of expression data (INMEX), a user-friendly web-based tool designed to support meta-analysis of multiple gene-expression data sets, as well as to enable integration of data sets from gene expression and metabolomics experiments. INMEX contains three functional modules. The data preparation module supports flexible data processing, annotation and visualization of individual data sets. The statistical analysis module allows researchers to combine multiple data sets based on *P*-values, effect sizes, rank orders and other features. The significant genes can be examined in functional analysis module for enriched Gene Ontology terms or Kyoto Encyclopedia of Genes and Genomes (KEGG) pathways, or expression profile visualization. INMEX has built-in support for common gene/metabolite identifiers (IDs), as well as 45 popular microarray platforms for human, mouse and rat. Complex operations are performed through a user-friendly web interface in a step-by-step manner. INMEX is freely available at http://www.inmex.ca.

## INTRODUCTION

High-throughput omics technologies have become commonplace in almost every biomedical research field. For any given disease or biological condition, there often exists a plethora of related data sets generated from the same or different omics platforms. With the emergence of public data repositories, such as the Gene Expression Omnibus (GEO) (1) and ArrayExpress (2), researchers have become increasingly interested in integrating their own data with the publicly available data sets to enable more accurate results and improved biological understanding.

Data integration can be classified into two general types—horizontal data integration and vertical data integration (3). Horizontal data integration, which is frequently used in meta-analysis, involves the combination and multi-facetted analysis of different data sets measuring the same molecular events under similar experimental conditions, for example, combining prostate cancer gene expression data sets from different studies. Vertical data integration, which is commonly used in systems biology, comprises combining data collected at different levels in the ‘omics-cascade’, for example, combining transcriptional and metabolic data sets from the same patient cohorts. Horizontal integration and meta-analysis of microarray data sets has been a field of intensive research in the past decade (3–17). Vertical integration of omics data has recently become a major focus in bioinformatics, as multi-level omics data sets (including single-nucleotide polymorphisms, gene, protein and metabolite expression data) are increasingly being collected from large clinical cohort studies (18–23).

A fundamental prerequisite for the integration of data sets from multiple studies is that all the data sets were collected under comparable experimental conditions, and/or the underlying experiments share the same hypothesis or are held to have the same mechanistic underpinnings. Horizontal data integration further requires the presence of the same or a significantly overlapping set of features (i.e. genes or probes) in each individual data set. There are several general steps in conducting microarray meta-analysis: (i) identification of suitable microarray studies and acquiring the data sets; (ii) annotation of each data set (including mapping different probe IDs to gene IDs, and ensuring phenotypic labels are accurate and consistent); (iii) combining data sets based on statistically rigorous methods; and (iv) analyzing and interpreting the results.

Given the increasing availability of public repositories, it is relatively straightforward to identify and determine whether a particular data set should be included or discarded. However, the challenges inherent in data management and the complexities of existing analytical approaches constitute two practical barriers to most bench biologists and clinicians interested in conducting data integration. These two issues have been tackled separately by two general approaches—database-oriented approaches and algorithm-oriented approaches. In the past few years, many microarray meta-analysis databases have been developed focusing on a particular disease or platform, including OncoMine (14), GeneSapien (24), Gemma (10), M^2^DB (11), CancerMA (25) and so forth. These databases are equipped with user-friendly web interfaces that allow researchers to view or query the gene expression profiles of pre-compiled microarray data sets. Conversely, the algorithm-oriented approach aims to provide robust methods to deal with the intrinsic variability and variation encountered during meta-analysis of microarray data. For instance, using the search term ‘meta-analysis’ on the popular Bioconductor portal will return a long list of R packages that have been implemented to perform various meta-analysis methods (26–30). The key idea is not to directly merge the original data sets but to combine them through high-level summary statistics, such as effect sizes, *P*-values and rank combinations to avoid the issues of inter-study variation (3). However, these two general approaches have limitations. In particular, the database-oriented approach depends on available meta-data (descriptions of what was done) and variability in the meta-data can limit decisions about which data should be combined. Similarly the use of algorithms in R packages requires significant bioinformatics skills, and it is especially difficult to compare different algorithms. User-friendly and flexible tools are still needed to assist researchers in performing these steps in an intuitive manner.

To address these needs, we have developed a web-based tool—INMEX (integrative meta-analysis of expression data). Our purpose is to provide an easy-to-use tool to assist bench biologists, clinicians and bioinformaticians in performing the common but convoluted procedures involved in omics data integration. INMEX’s main features include:

- Built-in support for common gene IDs and 45 popular microarray platforms;
- Built-in support for common metabolite names and major metabolomics database IDs;
- Flexible interface for uploading, processing and annotating individual data sets;
- Support for several well-established meta-analysis methods based on *P*-values, effect sizes, rank orders and vote counts, as well as direct data merging;
- Detailed result tables with summary statistics and gene-by-gene expression profiling;
- Clustering and visualizing the expression profiles of selected gene lists;
- Gene Ontology (GO) analysis;
- Pathway analysis and visualization.

INMEX is freely available at http://www.inmex.ca.

## PROGRAM DESCRIPTION AND METHODS

**Figure 1.**
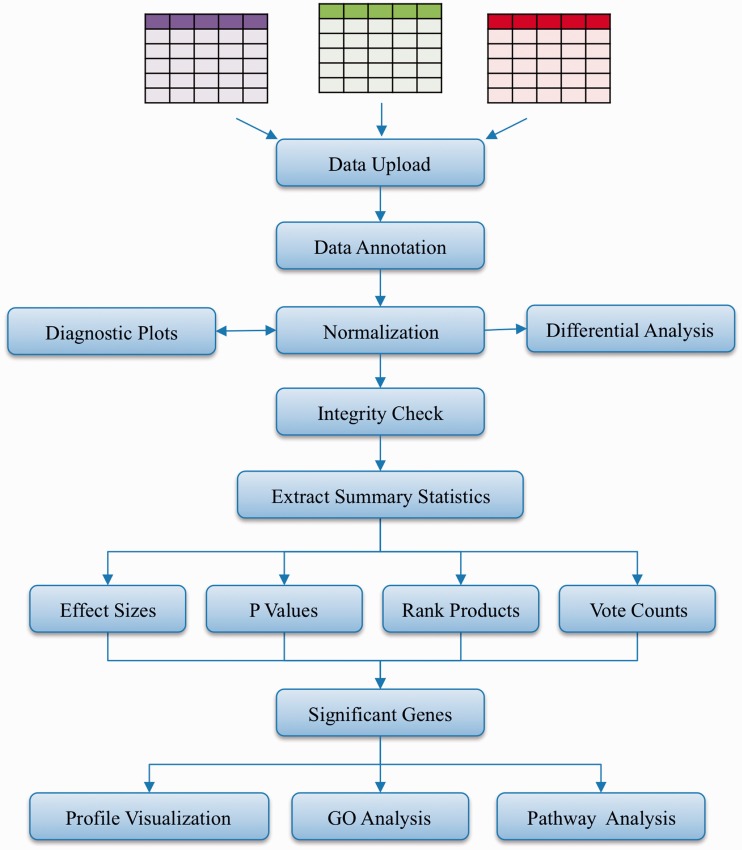
INMEX workflow. INMEX allows users to upload, process and annotate multiple gene expression data sets. After data integrity check, users can choose different methods to perform meta-analyses. The DE genes can be further visualized or examined for enriched GO terms or pathways.

INMEX uses a modular pipelined approach for analysis of expression data, a strategy that has proven popular among laboratory biologists (31–34). The overall workflow of INMEX is summarized in Figure 1. Briefly, the program is composed of three major modules corresponding to three sequential stages involved in the meta-analysis of gene expression data, namely, data preparation, statistical analysis and functional analysis. In addition, INMEX also contains a module for joint analysis of gene expression and metabolomics data. These modules are described in detail later in the text.

### Data preparation

**Figure 2.**
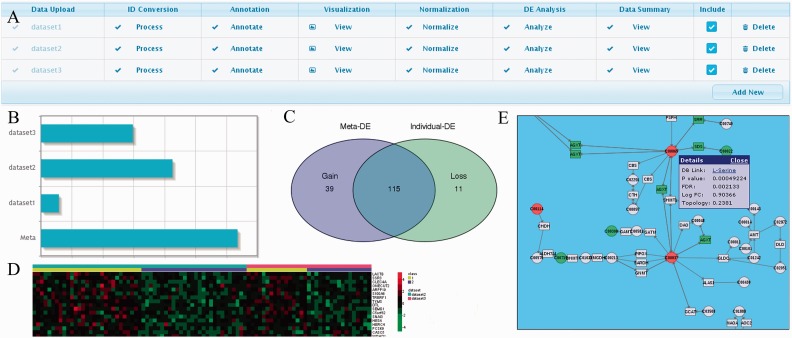
Some INMEX screenshots. (**A**) The table allows users to add, process, exclude or delete individual data sets for meta-analysis. Clicking on each table cell will trigger a dialog box to guide users through each step; (**B and C**) comparison of DE genes derived from meta-analysis and those based on individual data sets. (**D**) Heatmap visualization of a subset of genes across different studies; (**E**) visualization of significant genes and metabolites in a metabolic pathway. Red color indicates upregulation and green indicates downregulation. Clicking on each node will show more details.

The data preparation module allows users to upload and prepare multiple data sets for integration. This is a critical step and requires careful manual examination of each data set by users. To facilitate the process, we implemented a table-based navigation approach with eight columns corresponding to eight data processing steps as described later in the text. Adding a new row to the table will allow user to upload a new data set. Clicking on the cells within each row will bring up a series of dialog boxes that will guide users to complete the processing steps on the corresponding data. A screenshot is shown in Figure 2A with three data sets uploaded and processed. To control the computational load, the public server currently allows a maximum of 1000 samples to be uploaded and analyzed.

#### Data upload

The inputs for the data upload step are data tables containing gene expression values or relative expression values with genes/probes in rows and samples/experiments delineated in individual columns. The row containing the class labels for each sample (e.g. control, treatment and so forth) must be labeled with #CLASS in column 1. Multiple clinical parameters can be included in separate rows, for instance, #CLASS:cancer and #CLASS:treatment. The data table can be uploaded to INMEX as a tab delimited text file (.txt) or in a compressed file format (.zip). The ‘data formats’ page in INMEX websites contains detailed descriptions, as well as example data sets for illustration purposes.

#### Data annotation

After a data set is successfully uploaded, users need to annotate the data by converting different feature IDs to a common ID. They must also inspect the data to determine whether the same set of class labels is consistently used across different data sets.

##### Matching feature ID

This dialog box allows users to map different gene or probe IDs to Entrez IDs. When multiple probes are mapped to the same gene, the results will be presented as an average for combined probes. INMEX currently supports four types of common gene IDs (Entrez, RefSeq, GenBank and Ensembl) and 45 probe-set IDs corresponding to 45 microarray platforms for human, mouse and rat. For metabolites (in vertical integration analyses), all metabolite IDs will be converted to KEGG compound IDs. INMEX supports four major compound database IDs, as well as common compound names based on the Human Metabolome Database (HMDB) (35).

##### Matching class labels

This dialog box allows users to examine samples and their class labels for each data set. Users can change the class labels either in a batch manner, or by editing the labels for specific samples. Users can also remove specific samples via this dialog box.

#### Data visualization and normalization

The data visualization dialog box allows users to examine the quality of the current data set and to determine whether further data transformations are advisable and/or necessary. INMEX provides two diagnostic plots—box-and-whisker plots (boxplots) and principal component analysis scores plots. They help users identify outlier samples. INMEX expects all data values to be compared on a log2 scale with the same empirical distribution for each sample. The normalization dialog supports logs transformation and/or quantile normalization (36). Users can easily decide whether it is necessary to perform the procedure by visualizing the boxplots: log2 transformed data values should always be in the range of 0–16, and quantile normalized data should show identical distribution in the boxplots.

#### Differential expression analysis of individual data set

INMEX currently only considers the comparison between two conditions (defined by the class labels) for meta-analysis. When more than two conditions are present in the data sets, users need to specify which two conditions are to be compared. The moderated t-tests based on the Limma algorithm (37) are used to perform this analysis. Note, the results from individual data analyses are only for reference comparison and are not required for meta-analysis in the next stage.

#### Data integrity check

When all data sets have been uploaded, processed and annotated, users need to perform a data integrity check before proceeding to the meta-analysis stage. INMEX will inspect each individual data set to make sure that the class labels are consistent across all data sets and a significant number of common features can be identified. When inconsistencies are detected and flagged by INMEX, users can either edit the class labels using the ‘annotate’ functions as described before (in the case of discrepancies on class labels), or simply exclude the data set(s) (in the case of very few overlapping features or with too many missing values).

### Statistical analysis

Microarray data integration continues to be a challenging problem because of the inherent heterogeneity of individual data sets. Although many sophisticated algorithms have been published in recent years, no single statistical method is optimal. The method of choice depends on the underlying individual data quality, as well as the overall homogeneity of all data sets. For INMEX, we chose to implement several popular and robust approaches based on three main criteria: (i) ease of use and interpretation; (ii) ability to deal with different levels of data heterogeneity; and (iii) computational efficiency, which is important for a public web server. Based on these criteria, we implemented five different algorithms for meta-analysis. These approaches are briefly described later in the text.

#### Combining P-values

Calculating and combining *P*-values from multiple studies as a means of data integration has long been used in the meta-analysis of microarray data (6). This approach is simple to calculate and flexible to use. Fisher’s method and Stouffer’s method are two popular approaches. They have similar levels of performance and can be easily interpreted whereby larger scores reflect greater differential expression. The main difference is that weights (i.e. based on sample sizes) are incorporated into the calculation in Stouffer’s method, whereas Fisher’s method is known as a weight-free method. However, in microarray meta-analysis, larger sample size does not warrant larger weights, as the quality of each study can be variable. Users should choose to apply Stouffer’s method only when all studies are of similar quality (i.e. same platform with similar levels of missing values).

#### Combining standardized differences

Standardized difference, also known as effect size, is the difference between two group means divided by the standard deviation for the data (Cohen’s ‘d’). Standardized differences are considered combinable across studies. There are two popular methods to do this, namely, the fixed and random effects models (FEM and REM) (4). FEM assumes that there is one true effect size that underlies all studies in the analysis. In contrast, REM allows the true effect size to vary from study to study by incorporating unknown cross-study heterogeneities in the model (i.e. because of different platforms). Cochran’s Q test is commonly used to evaluate the homogeneity of the data sets (38). It is calculated as the weighted sum of the squared differences between individual study effects with the effects pooled across studies. INMEX implements a Q–Q plot to help users choose the appropriate models. When the estimated Q values have an approximately chi-squared distribution, the FEM assumption is most appropriate; otherwise, REM should be used. The implementation of this method is based on moderated effect size using the *metaMA* package (12).

#### Combining rank orders

One downside of combining *P*-values or standardized differences is that the results can often be affected by outliers, which are common in microarrays. Non-parametric approaches based on rank orders are more robust in this case. The implementation is based on the *RankProd* package as described by Hong *et al.* (26). Briefly, for each data set, the ratios (fold changes) are computed for all possible pairwise comparisons. The ranks of the ratios within each comparison are then used to calculate the rank product for each gene. Permutation tests are then performed to assess the null distributions of the rank product within each data set. The whole process repeats multiple times to compute *P*-value and false discovery rate (FDR) associated with each gene.

#### Combining votes

Vote counting is the most primitive but simplest and most intuitive method of meta-analysis. In the context of meta-analysis of microarray gene expression data, differential expression (DE) genes are first selected based on some criteria (e.g. adjusted *P* < 0.05) for each data set. The vote for each gene can then be calculated by counting the total number of times it occurs as DE across all data sets. This method is statistically inefficient and should be considered as a last resort in situations when other meta-analysis methods cannot be applied.

#### Direct data merging

In this approach, different data sets are merged into a mega-data set and then analyzed as if all data sets were derived from a single experiment. This approach ignores the inherent bias and heterogeneity of data sets from different sources. Many other factors (experiment protocols, technical platforms, raw data processing procedures and so forth) can potentially contribute to the observed differences. Therefore, this approach should only be used when data sets are similar (i.e. from the same platform without batch effects).

These algorithms described earlier in the text can deal with different levels of heterogeneity in the data sets. In particular, the direct merging method requires all data sets to be highly homogenous, combining *P*-values or standardized differences (i.e. using REM) can accommodate study-specific effects; the rank-based approach is more robust in the face of outliers and larger variations in different studies, whereas the vote counting method is essentially platform independent, as only the final DE gene lists are used. More technical details and instructions are provided as a comprehensive list of questions and answers in the ‘FAQs’ page on INMEX website. The results from meta-analysis are presented in detailed tables containing statistics from individual differential analyses, as well as statistics using the selected meta-analysis method. Users can click on any gene name to visualize its expression profiles across different studies. Figure 2B and C depicts the screenshots of high-level summaries of the DE genes identified from meta-analysis and those based on individual data sets.

### Functional analysis

INMEX’s functional analysis module is designed to help users generate new hypotheses by taking advantage of inherent characteristics of the DE gene list identified in the previous meta-analysis. ‘Pattern extractor’ enables users to choose a flexible set of genes and visualize their expression profiles across different data sets/conditions as heatmaps (Figure 2D); ‘GO analysis’ uses hypergeometric tests to allow the detection of enriched functional attributes (based on gene-associated GO terms) (39); ‘pathway analysis’ allows users to identify and visualize significantly enriched pathways based on the KEGG database. More advanced functional analysis, including network analysis and visualization, can be further pursed using InnateDB (40).

### Pathway-based integrative analysis and other utilities

This module gives INMEX a unique capacity to integrate two commonly encountered omics data sets—gene expression data and metabolomics data, based on the framework of KEGG pathways. Pathway creation and presentation was based on the R package *iSubpathwayMiner* (41). Users need to first upload both a gene expression data set and a metabolite concentration data set (‘Data Preparation’ section). The pathway analyses are performed in two steps. In the first step, significant genes and metabolites are identified from each corresponding data set; in the second step, these genes and metabolites are mapped to pathways for overrepresentation analysis and pathway topology analysis based on the concept that changes in both gene expressions and metabolite concentrations imply pathway involvement. The matched pathways can be visualized intuitively using a Google-map style pathway viewer (Figure 2E) (42). Users can switch between three modes for pathway analysis—a gene-and-metabolite mode (default), a gene-centric mode or a metabolite-centric mode. Unlike transcriptomic analyses, current metabolomics technologies capture only a partial metabolome and produce inherently biased results. The available options allow the user to explore results based on individual data sets.

INMEX also provides several utility tools to facilitate data operations commonly used in omics data integration. These include gene ID conversion, metabolite ID conversion and pathway mapping.

### Implementation, user data and session management

INMEX’s web interface was developed using the latest Java Server Faces 2.0 technology. The back end statistical computation and visualization were implemented using the R programming language. INMEX is designed to facilitate exploratory data analysis and real-time interaction with the users and is especially designed for biologists with modest computational skills. Results are returned in a few seconds to a few minutes. The most time consuming part is the data preparation stage, as for each individual data set uploaded, users need to go through the steps of processing, annotation and normalization. Once all data sets have been processed and pass the integrity check, the statistical and functional analysis can be performed efficiently.

Each time a user starts a session, a temporary account is created together with a temporary folder to store all user uploaded data sets and analytical results. Users are expected to download all their processed data sets, images and result tables on completion of a session. The data will remain on the server for 72 h and then is automatically deleted. For users who cannot complete all the analysis in one session, or want to explore the same data sets in future, they can save the processed data (INMEX_metadata.txt) from the current session, and re-upload this file to INMEX next time to avoid the time-consuming data preparation stage.

## CAVEATS AND LIMITATIONS

Meta-analysis is a complex task, and users need to be wary of many of the potential pitfalls and limitations. A fundamental requirement is that all data sets should be collected under highly similar experimental conditions and tissue types and contain control experiments. However, different investigators may use slightly different criteria in classifying positive and negative cases (e.g. using a chronic obstructive lung disease data set as a control for asthma). In addition, it is well known that different investigators can use different terms for the same condition or the same terms for different conditions, and these need to be rationalized based on the context and semantics. This information is usually in the form of free text in the publications or associated websites. Users are advised to pay close attentions to the meta-data (i.e. descriptions of each experiment), and if in doubt, contact the original authors for clarification.

INMEX currently performs probe to gene mapping based on their Entrez IDs. However, it is well known that the measured intensity of any probe depends not only on the target mRNA abundance but also on the sequence of the probe. Therefore, two arrays using different probe sequences for the same gene may not be directly comparable. A better approach is to BLAST all these probe sequences and only combine probes with substantial sequence overlap (43).

INMEX has been developed primarily to support data generated from popular commercial microarray platforms from Affymetrix GeneChip, Illumina BeadArray and Agilent one-color microarrays with common experimental designs for two group comparisons and multiple biological replicates. These commercial platforms are widely used, with more consistent quality and publicly available annotation information. INMEX currently does not support the analysis of data from complex (mostly in-house) two-color cDNA microarrays.

## COMPARISON WITH OTHER EXISTING TOOLS

As indicated by Tseng *et al.* (3), most tools developed for meta-analysis of microarray gene expression data sets are either databases with limited support to enable users to upload, annotate and analyze their own data sets, or they are algorithms that require significant bioinformatics skills to use. Nonetheless, several tools have been developed in the past few years that offer functionalities similar to INMEX, such as A-MADMAN (15), WGAS (17) and GEOSS (15). Table 1 shows a detailed comparison among these tools. As indicated in the table, the focus of these tools is more directed toward data management and raw data processing, whereas the meta-analysis method is either primitive or restricted to a single platform (i.e. Affymetrix). Substantial bioinformatics skills are required to set-up, maintain and customize these programs if other platforms are to be supported. In contrast, INMEX offers three distinctive features: (i) it focuses on gene expression data tables that are generally available from public repositories; (ii) it supports multiple advanced data integration algorithms to reduce study-specific effects; (iii) its interface is facile, interactive and easy to use. INMEX is designed for users with modest bioinformatics skills. For joint analysis of gene expression data and metabolomics data, INMEX complements other existing tools, such as Paintomics (21), IMPaLA (20) and Metascape (23) by providing a complete data analysis pipeline that enables data annotation, normalization and differential analysis through its user-friendly user interface, in addition to pathway-enrichment analysis and joint visualization of significant genes and compounds. The result tables downloaded from INMEX can also be used as input for the three tools aforementioned.

**Table 1.** Comparison with other microarray meta-analysis tools

Tool	INMEX	A-MADMAN	WGAS	GEOSS (GeneX)
Software type	Web-based (public server)	Web-based (install locally)	Web-based (public server)	Web-based (install locally)
License	Free	Free	Free (registry required)	Free
Platforms	Any single-channel platform	Affymetrix	Affymetrix	Affymetrix
Data input	Expression tables	CEL files	CEL files	CEL files
Raw data processing	N/A	Yes	Yes	Yes
Probe annotation	Yes (45 built-in)	Yes	Yes	Yes
Meta-analysis	*P*-values, effect sizes, rank products, vote counts, direct merge	Direct merge	Direct merge	Direct merge
Integration with other omics data	Yes (metabolomics)	No	No	No
Functional analysis	Yes	No	Yes	No
Data management	Basic no long-term storage	Yes	Yes	Yes

## CONCLUSIONS

In the past few years, many databases and algorithms have been developed for the meta-analysis of microarray data sets. However, user-friendly software tools to assist bench biologists and clinicians in performing meta-analysis are still lacking. In this article, we have presented INMEX, a web-based user-friendly tool for integrative meta-analysis of expression data to address this gap. INMEX supports facile data upload, flexible data annotation, comprehensive meta-analysis approaches, as well as integrative analysis of metabolomic and transcriptomic data. With the increasing numbers of data sets that are being generated and becoming publicly available, INMEX will become a valuable tool to the research community.

## FUNDING

Funding to support this research was obtained from the Canadian Institutes for Health Research. J. Xia was supported by a Canadian Institutes of Health Research Postdoctoral Fellowship and Killam Postdoctoral Research Fellowship. REWH holds a Canada Research Chair. Funding for open access charge: Canadian Institutes of Health Research.

*Conflict of interest statement*. None declared.
